# Supplementary material for: Systematic Identification of Gene Families for Use as “Markers” for Phylogenetic and Phylogeny-Driven Ecological Studies of Bacteria and Archaea and Their Major Subgroups
Source: PLoS One. 2013 Oct 17;8(10):e77033. doi: 10.1371/journal.pone.0077033 (PMC3798382; doi:10.1371/journal.pone.0077033)
Supplement: Table S1 — Summary of 74 PhyEco marker candidates identified for the group “Bacteria”. (DOC) [file pone.0077033.s001.doc]

Table S1: Summary of 74 PhyEco marker candidates identified for the group “Bacteria”.

| Marker ID | Gene Family Descriptions | Correspondent AMPHRA-I Marker |
| --- | --- | --- |
| B000041 | transcription elongation protein NusA | nusA |
| B000042 | rpoB DNA-directed RNA polymerase subunit beta | rpoB |
| B000043 | GTP-binding protein EngA | - |
| B000044 | rpoC DNA-directed RNA polymerase subunit beta' | - |
| B000045 | priA primosome assembly protein | - |
| B000046 | transcription-repair coupling factor | - |
| B000047 | CTP synthase | pyrG |
| B000048 | secY preprotein translocase subunit SecY | - |
| B000049 | GTP-binding protein Obg/CgtA | - |
| B000050 | DNA polymerase I | - |
| B000051 | rpsF 30S ribosomal protein S6 | - |
| B000052 | poA DNA-directed RNA polymerase subunit alpha | - |
| B000053 | peptide chain release factor 1 | - |
| B000054 | rplI 50S ribosomal protein L9 | - |
| B000055 | polyribonucleotide nucleotidyltransferase | - |
| B000056 | tsf elongation factor Ts | tsf |
| B000057 | rplQ 50S ribosomal protein L17 | - |
| B000058 | tRNA (guanine-N(1)-)-methyltransferase | rplS |
| B000059 | rplY probable 50S ribosomal protein L25 | - |
| B000060 | DNA repair protein RadA | - |
| B000061 | glucose-inhibited division protein A | - |
| B000062 | Unknown protein | - |
| B000063 | ribosome-binding factor A | - |
| B000064 | DNA mismatch repair protein MutL | - |
| B000065 | smpB SsrA-binding protein | smpB |
| B000066 | N-acetylglucosaminyl transferase | - |
| B000067 | S-adenosyl-methyltransferase MraW | - |
| B000068 | UDP-N-acetylmuramoylalanine--D-glutamate ligase | - |
| B000069 | rplS 50S ribosomal protein L19 | - |
| B000070 | rplT 50S ribosomal protein L20 | rplT |
| B000071 | ruvA holliday junction DNA helicase | - |
| B000072 | ruvB Holliday junction DNA helicase B | - |
| B000073 | serS seryl-tRNA synthetase | - |
| B000074 | rplU 50S ribosomal protein L21 | - |
| B000075 | rpsR 30S ribosomal protein S18 | - |
| B000076 | DNA mismatch repair protein MutS | - |
| B000077 | rpsT 30S ribosomal protein S20 | - |
| B000078 | DNA repair protein RecN | - |
| B000079 | frr ribosome recycling factor | frr |
| B000080 | recombination protein RecR | - |
| B000081 | protein of unknown function UPF0054 | - |
| B000082 | miaA tRNA isopentenyltransferase | - |
| B000083 | GTP-binding protein YchF | - |
| B000084 | chromosomal replication initiator protein DnaA | - |
| B000085 | dephospho-CoA kinase | - |
| B000086 | 16S rRNA processing protein RimM | - |
| B000087 | ATP-cone domain protein | - |
| B000088 | 1-deoxy-D-xylulose 5-phosphate reductoisomerase | - |
| B000089 | 2C-methyl-D-erythritol 2,4-cyclodiphosphate synthase | - |
| B000090 | fatty acid/phospholipid synthesis protein PlsX | - |
| B000091 | tRNA(Ile)-lysidine synthetase | - |
| B000092 | dnaG DNA primase | dnaG |
| B000093 | ruvC Holliday junction resolvase | - |
| B000094 | rpsP 30S ribosomal protein S16 | - |
| B000095 | Recombinase A recA | - |
| B000096 | riboflavin biosynthesis protein RibF | - |
| B000097 | glycyl-tRNA synthetase beta subunit | - |
| B000098 | trmU tRNA (5-methylaminomethyl-2-thiouridylate)-methyltransferase | - |
| B000099 | rpmI 50S ribosomal protein L35 | - |
| B000100 | hemE uroporphyrinogen decarboxylase | - |
| B000101 | Rod shape-determining protein | - |
| B000102 | rpmA 50S ribosomal protein L27 | rpmA |
| B000103 | peptidyl-tRNA hydrolase | - |
| B000104 | translation initiation factor IF-3 | infC |
| B000105 | UDP-N-acetylmuramyl-tripeptide synthetase | - |
| B000106 | rpmF 50S ribosomal protein L32 | - |
| B000107 | rplL 50S ribosomal protein L7/L12 | rpIL |
| B000108 | leuS leucyl-tRNA synthetase | - |
| B000109 | ligA NAD-dependent DNA ligase | - |
| B000110 | cell division protein FtsA | - |
| B000111 | GTP-binding protein TypA | - |
| B000112 | ATP-dependent Clp protease, ATP-binding subunit ClpX | - |
| B000113 | DNA replication and repair protein RecF | - |
| B000114 | UDP-N-acetylenolpyruvoylglucosamine reductase | - |
